# 2022 Reviewer Index

**DOI:** 10.26508/lsa.202301921

**Published:** 2023-01-23

**Authors:** Eric Sawey

**Affiliations:** Executive Editor, Life Science Alliance , Cold Spring Harbor, NY, USA

## Abstract

The LSA Editors thank these scientists for sharing their time and expertise while reviewing manuscripts in 2022. We sincerely appreciate their support.

Abdel-Rahman, Engy

Abu-Baker, Aida

Ackley, Brian D.

Adams, David

Adams, Josephine C.

Adhikari, Hema

Adrover, Jose M.

Ager, Ann

Aidinis, Vassilis

Aihara, Hideki

Akey, Christopher W.

Akondy, Rama S.

Aktories, Klaus

Alarcon, Balbino

Alexander, Matthew

Ali, Safdar

Allred, David R.

Almeida, Miguel

Almstrup, Kristian

Alushin, Gregory

Amberg, Nicole

An, Songon

Anand, Paras K.

Anderssen, Endre

Anjo, Sandra I.

Apostolou, Effie

Ariel, Federico

Armache, Jean-Paul

Arpağ, Göker

Assis, Raquel

Atreya, Imke

Autexier, Chantal

Azem, Abdussalam

Böhm, Volker

Böhmer, Frank D.

Böttcher, Ralph T.

Bøe, Stig O.

Babitzke, Paul

Bachant, Jeff

Bachmair, Andreas

Baes, Myriam

Baeza Garcia, Alvaro

Bagelova, Silvia

Bahadur, Ranjit

Baker, Kristian E.

Baldwin, Albert S.

Balla, Tamas

Balmus, Gabriel

Ban, Young H.

Banerjee, Sourav

Barau, Joan

Bardwell, James

Bardy, Cedric

Barrio Hernandez, Inigo

Barrios, Arantza

Baskakov, Ilia V.

Basler, Konrad

Basu, Uttiya

Baugh, Ryan

Baulida, Josep

Bayliss, Richard

Bebenek, Kasia

Becker, Peter B.

Bedford, Mark T.

Beilke, Joshua

Bekker-Jensen, Simon

Bellei, Barbara

Belser, Jessica

Bendahmane, Abdelhafid

Beningo, Karen

Benkovic, Steven J. J.

Bennett, Eric J.

Bertolin, Giulia

Beyer, Andreas

Bharat, Tanmay A.

Bhatia, Sonam

Bi, Erfei

Bierings, Ruben

Birney, Ewan

Birsa, Nicol

Birsoy, Kivanc

Biryukova, Inna

Bishop, Douglas K.

Blankenfeldt, Wulf

Blum, Robert

Bochkaryova, Olga

Bolli, Niccolo

Bonilha, Vera L.

Borys, Agnieszka M.

Bosis, Eran

Boucher, Dave

Boutet, Marie

Bowerman, Melissa

Boxer, Lisa

Brandenberger, Christina

Brandt, Roland

Branzei, Dana

Brar, Gloria

Braun, Ralf J.

Breunig, Joshua J.

Brill, Julie A.

Brindley, David N.

Brown, Andrew J.

Brown, Nick

Brummer, Tilman

Buée, Luc

Bubb, Kristen

Buratti, Emanuele

Burstein, Ezra

Busskamp, Volker

Bussolino, Federico

Caimi, Karina

Calin, George A.

Campaner, Stefano

Cao, Cun-Wei

Cao, Wei

Capitanio, Nazzareno

Cardozo, Christopher

Carnero, Amancio

Carreno, Sébastien

Carrer, Alessandro

Carreras-Sureda, Amado

Carrier, France

Carrillo, Jorge

Carroll, Bernadette

Carroll, Christopher

Carver, Brett S.

Cassatella, Marco A.

Castañeda, Carlos A.

Castaldi, Peter

Ceppi, Paolo

Cerutti, Andrea

Chávez-Gutiérrez, Lucía

Chacinska, Agnieszka

Chai, Zuying

Chakrabarti, Rajarshi

Chanfreau, Guillaume

Chao, Luke

Chapoval, Svetlana P.

Chappe, Valerie M.

Chappert, Pascal

Chaudhuri, Jayanta

Chen, Jiandong

Chen, Jun

Chen, Lingling

Chen, Lu

Chen, Song

Chen, Xuemei

Cherry, Amy

Cherry, Joshua L.

Cheung, Alan

Chevet, Eric

Chien, Wade W.

Chiocca, Susanna

Chiu, Joanna C.

Cho, Hee C.

Chopin, Michael

Christofori, Gerhard

Chung, Ka Young

Churchman, L. Stirling S.

Ciccolini, Francesca

Cingaram, Pradeep

Claessens, Antoine

Claesson, Hans-Erik

Clague, Michael J.

Claringbould, Annique

Clark, David J.

Clemmensen, Christoffer

Cobb, Melanie H.

Cohen, Philipp

Cohen-Fix, Orna

Colonna, Marco

Compton, Alex A.

Conticello, Silvestro G.

Contreras, Osvaldo

Cook, Matthew C.

Cooper, Thomas A.

Copeland, Paul

Copeland, William C.

Coraux, Christelle

Costanzo, Vincenzo

Cota, Damita

Coulombe, Pierre A.

Cox, Brian J.

Crepaldi, Tiziana

Crespo, Piero

Cui, Rutao

Cullen, Peter J.

Cunha, Thiago M.

Cvetanovic, Marija

Dalal, Yamini P.

Dantuma, Nico P.

Das, Maitreyi

Das, Rudra N.

Daum, Bertram

Day, Catherine L.

De Carvalho, Braulio F.

De Gruijl, Tanja D.

De la Luna, Susana

De Matteis, Antonella

De Oliveira, Tiago

De Souza, Robson Francisco

Decker, Thomas

Degterev, Alexei

Delamarre, Lelia

DELAVAL, Benedicte

Demarco, Rafael

Demeulemeester, Jonas

Den Brave, Fabian

Denisov, Ivan

Desingu Rajan, Ayyappa Raja

Dewson, Grant

Di, Li-Jun

Dibner, Charna

Diefenbacher, Markus

Dimitriadi, Maria

Dineley, Kelly T.

Ding, Chen

Dixon, Scott J.

Dobric, Aurelie

Dohmen, R. Jürgen

Dolezal, Pavel

Dominguez-Sola, David

Draviam, Viji

Drew, David

Driver, John

Drosten, Matthias

Duffy, Darragh

Duharcourt, Sandra

Dultz, Elisa

Dupuis, Luc

DYKHUIZEN, EMILY C.

Dyson, Jennifer M.

Dziembowski, Andrzej

E, Lezi

Ebert, Peter

Eisenman, Robert N.

El Fatimy, Rachid

Elfatih, Amal

Elgui de Oliveira, Deilson

Elias, Kevin

Elkon, Keith

Ellison, Christopher

Emanuele, Michael J.

Emperador-Melero, Javier

Engevik, Mindy

Epand, Richard M.

Eriksson, Ove

Erwin, Jennifer

Escobar-Henriques, Mafalda

Estella, Carlos

Ettensohn, Charles A.

Evans, Ian

Eyers, Patrick A.

Faessler, Reinhard

Fajkus, Jiri

Fandrey, Joachim

Fang, Evandro F.

Farnung, Lucas

Fearon, Douglas T.

Feliciello, Isidoro

Fellows, Alex

Fendt, Sarah-Maria

Fernandez-Chacon, Rafael

Fielitz, Jens

Firat Karalar, Elif N.

Firon, Arnaud

Fischer, Andre

Flak, Jonathan

Foley, Ann

Forst, Christian V.

Franken, Gijs

Fraser, Alasdair R.

Fratti, Rutilio A.

Freund, Patrick

Frey, Mark R.

Friedmann Angeli, José P.

Frischknecht, Friedrich

Frumkin, Idan

Fry, Andrew M.

FUCHS, Julia

Fujita, Masatoshi

Fustin, Jean-Michel

Futter, Clare E.

Götz, Magdalena

Gabellini, Davide

Gagliardi, Paolo

Gahan, James

Gallo, Jean-Marc

Gangwani, Laxman D.

Ganley, Ian G.

Ganz, Julia

Gao, Feng

Garcia, Sarela

Gartenberg, Marc R.

Gasser, Susan M.

Gavis, Elizabeth R.

Geijtenbeek, Teunis B.

Gems, David

Gerber, Scott

Gerber, Scott A.

Gerdes, Patricia

Gergely, Fanni

Gerton, Jennifer L.

Gewurz, Benjamin E.

Gilbert-Jaramillo, Javier

Gilkerson, Robert

Giron, Jorge A.

Gladkova, Christina

Goldberg, Alfred L.

Goldberg, Andrew F.

Goncalves, Emanuel

Goodman, Joel M.

Goodman, Myron F.

Gopalakrishnan, Jay

Graef, Martin

Gresham, David J.

Griffin, Ricarda

Grifoni, Alba

Grillet, Nicolas

Grimsholm, Ola

Grosche, Antje

Groschner, Lukas

Gross, Alecia K.

Gross, Christina

Gruenberg, Jean

Gruss, Oliver J.

Guigo, Roderic

Guillamat-Prats, Raquel

Guizetti, Julien

Gullberg, Donald

Gunning, Peter W.

Guo, Dongsheng

Gursky, Jan

Gyrd-Hansen, Mads

Hörnberg, Hanna

Haase, Astrid

Habib, Amyn A.

Haeusler, Aaron

Hamada, Mayuko

Hamilton, Kathryn E.

Hanigan, Thomas

Hanin, Aurelie

Hannun, Yusuf A.

Hantschel, Oliver

Hariri, Hanaa

Harms, Christoph

Harterink, Martin

Hartl, Ingrid

Hartley, Paul

Harton, Jonathan A.

Hasbi, Ahmed

Hashimoto, Hiroshi

Hattori, Motoyuki

Hayrabedyan, Soren B.

Hehir-Kwa, Jayne

Heineke, Joerg

Heinig, Matthias

Helgason, Vignir

Hempel, Georg

Hendricks, Adam G.

Hennig, Janosch

Herlyn, Meenhard

Hermey, Guido

Hernandez, Ciria C.

Herrmann, Johannes

Hilbert, Lennart

Hildebrandt, Friedhelm

Hilligan, Kerry

Hiraoka, Yasushi

Hirrlinger, Johannes

Hirsch, Joel A.

Hitoshi, Seiji

Hlocik, Martin

Hoffmann, Ary Anthony

Hornstein, Eran

Horovitz, Amnon

Hsia, Kuo-Chiang

Hsiao, Kuei-Yang

Huang, Alex

Huang, Lan

Huang, Niu

Huang, Yadong

Hunter, Tony

Hynds, Robert E.

Hynes, Richard O.

Iben, Sebastian

Ichtchenko, Konstantin

Ihrie, Rebecca A.

Ikeda, Yoko

Ikui, Amy E.

Imamura, Takuya

Ito, Toshihiro

Ittner, Lars M.

Izumi, Tadahide

Jégou, Antoine

Jékely, Gáspár

Jackson, Mark W.

Jacobs, Jacqueline J.

Jae, Lucas

Jaiswal, Jyoti

Jalkanen, Sirpa

Jans, David A.

Jarvis, Erich

Jensen, Robert

Jensen, Ryan

Jho, Eek-hoon

Jochmans, Dirk

Johansen, Terje

Johnson, Aaron

Johnson, Kenneth A.

Johnstone, Scott

Jonas, Elizabeth A.

Jontes, James

Joshi, Amit

Köster, Mario

Kabani, Mehdi

Kai, Toshie

Kajimura, Shingo

Kakumani, Pavan K.

Kang, Shan

Kaplan, Craig D.

Kapranov, Philipp

Kapuria, Vaibhav

Katara, Pramod

Kay, Robert R.

Kermorgant, Stephanie

Ketting, Rene F.

Khalimonchuk, Oleh

Khamlichi, Ahmed A.

Khan, Muzamil

Khan, Wasim

Kiebler, Michael A.

Kietzmann, Thomas

Kim, Jonathan

Kim, Seon-Young

Kim, Wantae

Kinesberger, Petra C

Kirsch, David G.

Kissler, Stephan

Klein, William L.

Kleinridders, Andre

Koffler, Jacob

Kojima, Kenji K.

Koller, Dora

Kolmogorov, Mikhail

Komander, David

Komatsu, Masanobu

Kong, Daochun

Kong, Zhaosheng

Koralov, Sergei B.

Korkhov, Vladimir M.

Kornberg, Thomas B.

Kornitzer, Daniel

Kouznetsova, Anna

Krämer, Helmut

Kröger, Andrea

Kraft, Claudine

Krappmann, Daniel

Krasnitz, Alexander

Krauss, Michael

Krebs, Stefan

Kreienkamp, Hans-Jürgen

Krestel, Heinz E.

Krueger, Elke

Krueger, Stefan

Krzycki, Joseph

Kumar, Manish

Kumari, Pooja

Kumsta, Caroline

Kurose, Hitoshi

López-Farfán, Diana

La Cava, Antonio

Lacy, D. B.

Lada, Artem

Lafontaine, Denis L.

Lahaye, Xavier

Laidlaw, Brian J.

Lambert, Nevin A.

Lambrecht, Bart N.

Langlois, Ryan A.

LaRock, Christopher N.

Lattanzi, Giovanna

Leach, Lyndsay

Leclercq, Georges

Lee, Chih-Hao

Lee, Kai-Fei

Lee, Tina H.

Leeb, Martin

Lentz, Jennifer

Lescure, Alain

Leung, Nelson

Leushkin, Evgeny

Li, Jingxian

Li, Lijia

Li, Xin

Li, Zhiguo

Liebscher, Gudrun

Lihrmann, Isabelle

Lin, Chen-Ching

Lingner, Joachim

Link, Christopher D.

Lishko, Polina V.

Liu, Fei

Liu, Ru-Juan

Llorente, Bertrand

Lopez, Bernard S.

Lotem, Michal

Ludwig, Bernd

Lugli, Gabriele Andrea

Lukens, John

Lum, Julian J.

Lusk, C P.

Lykke Lind, Barbara

Münch, Christian

Ma, Jiyan

Mabb, Angela

MacGillavry, Harold

Macleod, Kay F.

MADER, Sylvie

Magin, Thomas M.

Maire, Pascal

Makalowska, Izabela

Mammoto, Akiko

Manadas, Bruno

Mancilla-Galindo, Javier

Mange, Alain

Mannervik, Mattias

Manoury, Benedicte B.

Marashi, Sayed Amir

Marchionni, Luigi

Marfany, Gemma

Mark, Brian L.

Markert, Elke

Martinez, Aitor

Martinez-Salas, Encarnacion

Marx, Andreas

Marygold, Steven

Masai, Hisao

Mashek, Doug

Masuda, Takahiro

Mata, Juan

Mattevi, Andrea

Matthews, Paul M

Mavrothalassitis, George

Mayerhofer, Artur

McCann, Chase

McCaughan, Frank

McWilliam, Hamish

Meagher, Thomas

Meakin, Ashley

Medin, Jeffrey A.

Melichar, Heather J.

Mer, Georges

Miceikaite, Ieva

Millar, David

Mine, Judith

Mirabelli, Carmen

Mitra, Abhisek

Moffat, Jason

Mohammed, Altaf

Molkentin, Jeffery D.

Momburg, Frank

Morano, Kevin A.

Morrison, Ciaran

Moser, Tobias

Mozaffari-Jovin, Sina

Muehleder, Severin

Mueller, Ferenc

Muench, Stephen P.

Mukherjee, Rukmini

Murayama, Kazutaka

Musselman, Catherine

Mylne, Joshua

Mymryk, Joe S.

Nakatsu, Fubito

Nakayama, Keiko

Navaratnarajah, Chanakha K.

Navarro, Pablo

Neiman, Aaron M.

Neogi, Ujjwal

Ng, Lai G.

Nguyen, Long V.

Nishida, Eisuke

O'Carroll, Donal

O'Farrelly, Cliona

Öhlund, Daniel

Olafsson, Gudjon

Oltean, Sebastian

Öncül, Begüm Gençer

Onn, Itay

Osley, Mary A.

Osman, Christof

Ostrom, Rennolds

Ou, Guangshuo

Oz, Hasan

Ozisik, Ozan

Padmanabhan, Krishnan

Palstra, Robert-Jan

Pangas, Stephanie

Parast, Mana

Parastesh, Mohamad

Pardo, Benjamin

Parent, Jack

Paronetto, Maria Paola P.

Partridge, Linda

Parts, Leopold

Pasqualini, Renata

Patil, Kiran

Patrignani, Paola

Pattini, Linda

Paul, Soumen

Pedemonte, Nicoletta

Pedersen, Stine F.

Peraza-Reyes, Leonardo

Perrakis, Anastassis

Perrimon, Norbert

Pessolano, Emanuela

Petter, Michaela

Picard, Martin

Pin, Christopher L.

Pintard, Lionel

Pishas, Kathleen I.

Plaschka, Clemens

Poelzing, Steven

Polesello, Cedric

Polesskaya, Anna

Polishchuk, Roman

Pollard, Luther

Poulton, John S.

Powell, Jennifer

Powell, Rebecca

Powell, Simon N.

Prabhu, Sumanth D.

Prajapati, Hemant

Prekeris, Rytis

Prinz, Immo

Prinz, William

Pruneda, Jonathan N.

Pujol, Aurora

Puri, Pier L.

Qiao, Liang

Qu, Xiaoyu

Quattrocelli, Mattia

Quiding-Järbrink, Marianne

Rao, Anjana

Rao, Rajini R.

Rauchman, Michael

Remenyi, Attila

Reth, Michael

Rethi, Bence

Reyes Palomares, Armando

Reynolds, Nicholas

Rhind, Nicholas

Riley, Paul R.

Rittinger, Katrin

Robertson, Erle S.

Roca, Xavier

Rocha, Sonia

Roddy, Gavin

Roh, Soung-Hun

Romani, Andrea M.

Romanin, Christoph

Romero, Héctor

Rosenfeld, Michael G.

Rosina, Marco

Rothlin, Carla

Royle, Stephen J.

Rubio, Karla

Rudnicki, Michael A.

Runge, Kurt

Rutkowski, Joseph

Ryzhov, Sergey

Sánchez-Madrid, Francisco

Saada, Ann

Saccone, Valentina

Sahin, Umut

Sahu, Parul

Saito, Yoshiro

Sanyal, Mrinmoy

Scherer, Erin

Scherz-Shouval, Ruth

Schick, Sandra

Schinder, Alejandro

Schlevogt, Bernhard

Schmidt, Alex

Schroeder, Bernd

Schubeler, Dirk

Schuck, Sebastian

Schultz, Patrick

Schultze-Hentrich, Julia

Schumacher, Michael A.

Schwaiger, Michaela

Schwartz, Thomas U.

Schwarz, Thomas L.

Semczuk, Andrzej

Sena de Souza, Janaina

Sengupta, Abhishek

Sevcikova, Sabina

Seydoux, Geraldine

Sferruzzi-Perri, Amanda

Shang, Jinsai

Shao, Ping Y.

Sharif, Omar

Sheikh-Hamad, David

Shelburne, Samuel A.

Shen, Cailiang

Sheridan, Brian S.

Shih, Hsiu-Ming

Shilpa, Buch

Shim, Hyunbo

Shindou, Hideo

Shirihai, Orian S.

Shirinian, Margret

Shtrahman, Matthew

Shtutman, Michael

Shu, Yilai

Shukla, Vinay

Shuster, C. Brad

Shutt, Timothy E.

Sikela, James

Silke, John

Silvestre Roig, Carlos

Silvin, Aymeric

Singh, Rakesh

Sivak, Jeremy M.

Skvortsova, Ksenia

Skwark, Marcin J.

Sleigh, James N.

Soller, Matthias

Soppa, Jörg

Spence, Jeff

Sreelatha, Anju

Srinivasan, Madhu

Srinivasan, Sathish

Stasiak, Andrzej

Stecca, Barbara

Stegemann-Koniszewski, Sabine

Stein, Jason

Steingrímsson, Eiríkur

Steinmetz, Lars

Steller, Hermann

Stellos, Konstantinos

Strasser, Geraldine

Suetsugu, Shiro

Sun, Yang

Sundborger-Lunna, Anna

Suo, Satoshi

Sutterwala, Fayyaz S.

Suzuki, Yutaka

Svoboda, Petr

Szalai, Ben

Takeda, Makoto

Tan, Aimee

Tan, Junjie

Tanaka, Naoki

Tantin, Dean

Taraska, Justin W.

Tavassoli, Ali

TCW, Julia

Teis, David

Telang, Sucheta

Telange, Rahul

Tessarz, Peter

Thompson, Barry J.

Thomsen, Hauke

Thornell, Ian

Tikkanen, Ritva

Tilley, Leann

Tilmont, Rémi

Timchenko, Nikolai

Titus, Margaret A.

Toda, Takashi

Tokatlidis, Kostas

Tomasetto, Catherine L.

Tooze, Sharon A.

Toshchakov, Vladimir Y.

Tournier, Cathy

Toussaint, Ariane

Towers, Greg J.

Tran, Pamela

Trotti, Davide

Tuma, Pamela L.

Tyson, Darren R.

Tzfati, Yehuda

Ullah, Zakir

Ullrich, Susanne

Updike, Dustin

Uribe, Rosa A.

Vaiman, Daniel

Valcarcel, Lorea

Valdmanis, Paul N.

Valiente-Echeverría, Fernando

Vamosi, Gyoergy

Van den Boom, Johannes

Van der Does, Anne

Van Egmond, Marjolein

Van Strijp, Jos A.

Vergara, Natalia

Vermeesch, Joris R.

Vermeulen, Michiel

Vicente-Manzanares, Miguel

Vigneron, Arnaud

Vignoli, Beatrice

Vinkemeier, Uwe

Vischi Winck, Flavia

Von Eckardstein, Arnold

Von Lintig, Johannes

Vucic, Domagoj

Vukušić, Kruno

Wahl, Markus

Wai, Timothy

Walker, David

Walko, Gernot

Wallace, Helen

Wang, Cheng-I

Wang, Huating

Wang, Lianchun

Wang, Tianyi

Wang, Weiwei

Wang, Xinquan

Wang, Xiu-Jie

Wang, Yidong

Wang, Yifan

Wang, Yong-Qin

Wang, Zhong

Warnecke, Tobias

Warren, Alan J.

Wei, Lai

Wei, Zhiyun

Weidberg, Hilla

Weingartner, Magdalena

Weinhofer, Isabelle

Weir, Gordon C.

Wells, Timothy

Wen, Weihong

Werth, Victoria

West, Ryan J. H.

Westaway, David

Westmark, Cara J.

Whitsett, Jeffrey A.

Whittaker, Gary R.

Wiest, David L.

Wiley, H. S.

Wilk, Sherwin

Wilkinson, Miles F.

Willnow, T E.

Winkler, Bettina

Winter, Georg E.

Wiseman, Luke

Withey, Jeffrey

Wong, Amy P

Wright, Stephanie C.

Wu, Hao

WU, Zhe

Xiao, Tianshu

Xu, Chao

Xu, Mengyang

Xu, Pingwen

Yamamoto, Masahiro

Yamamoto, Yusuke

Yan, Shun

Yang, Changqing

Yang, Chenglin

Yang, Jing

Yao, Changfu

Yao, Leong Jing

Yin, Shen

Yin, Shiyi

Ying, Zheng

Yipp, Bryan

Yount, Jacob

Yu, Luyang

Yue, Jessica

Yumura, Shigehiko

Zacarias-Fluck, Mariano F.

Zaidel-Bar, Ronen

Zanetti, Maurizio

Zarkada, Georgia

Zauderer, Maurice

Zechner, Ellen

Zeng, Gucheng

Zeng, Lingchan

Zha, Xiaohui

Zhang, Jian

Zhang, Jingyuan

Zhang, Min

Zhang, Si-Ming

Zhao, Le

Zheng, Lei

Zheng, Lei

Zhou, Xiaofeng

Zhou, Zhaolan

Zissel, Gernot

Zoladek, Teresa

Zwick, Michael B.

